# Methionine Restriction Activates the Retrograde Response and Confers Both Stress Tolerance and Lifespan Extension to Yeast, Mouse and Human Cells

**DOI:** 10.1371/journal.pone.0097729

**Published:** 2014-05-15

**Authors:** Jay E. Johnson, F. Brad Johnson

**Affiliations:** 1 Department of Pathology and Laboratory Medicine, University of Pennsylvania, Philadelphia, Pennsylvania, United States of America; 2 Institute on Aging, University of Pennsylvania, Philadelphia, Pennsylvania, United States of America; University of Washington, United States of America

## Abstract

A methionine-restricted diet robustly improves healthspan in key model organisms. For example, methionine restriction reduces age-related pathologies and extends lifespan up to 45% in rodents. However, the mechanisms underlying these benefits remain largely unknown. We tested whether the yeast chronological aging assay could model the benefits of methionine restriction, and found that this intervention extends lifespan when enforced by either dietary or genetic approaches, and furthermore, that the observed lifespan extension is due primarily to reduced acid accumulation. In addition, methionine restriction-induced lifespan extension requires the activity of the retrograde response, which regulates nuclear gene expression in response to changes in mitochondrial function. Consistent with an involvement of stress-responsive retrograde signaling, we also found that methionine-restricted yeast are more stress tolerant than control cells. Prompted by these findings in yeast, we tested the effects of genetic methionine restriction on the stress tolerance and replicative lifespans of cultured mouse and human fibroblasts. We found that such methionine-restricted mammalian cells are resistant to numerous cytotoxic stresses, and are substantially longer-lived than control cells. In addition, similar to yeast, the extended lifespan of methionine-restricted mammalian cells is associated with NFκB-mediated retrograde signaling. Overall, our data suggest that improved stress tolerance and extension of replicative lifespan may contribute to the improved healthspan observed in methionine-restricted rodents, and also support the possibility that manipulation of the pathways engaged by methionine restriction may improve healthspan in humans.

## Introduction

It is well documented in rodents that a diet with a normal caloric content, but containing limiting amounts of methionine, robustly improves healthy lifespan. Rats fed such a diet are up to 45% longer-lived than control rats [1], [2]. Methionine-restricted mice benefit from a less robust, but still significant extension of lifespan and show a marked amelioration of various age-related pathologies as compared with mice fed a normal diet [3]. While the mechanistic basis of this benefit remains largely unknown, it has been suggested that methionine restriction (Meth-R) might act through mechanisms as diverse as reducing the rate of translation, altering gene expression through hypomethylation of nucleic acids, inducing stress hormesis, modulating the levels of glutathione or endocrine factors like IGF-1, or limiting the production of reactive oxygen species (ROS) [2]–[6].

A clue to the mechanistic basis of Meth-R might be found, however, in the observation that cellular stress resistance tends to correlate positively with cellular and organismal longevity. For example, similar to Meth-R, rapamycin treatment robustly extends lifespan in mammals [7], [8], and TOR (‘Target Of Rapamycin’, which is inhibited by rapamycin) negatively affects stress tolerance [9]–[11]. In addition, skin-derived fibroblasts from long-lived mouse strains are resistant to a number of cytotoxic stresses [12]–[14]. Collectively, such findings raise the possibility that interventions that confer organismal lifespan extension, like Meth-R, might do so by improving cellular stress tolerance.

To study the underlying basis of lifespan extension by Meth-R, we developed genetically tractable cell-based model systems. The first of these, the yeast chronological aging assay, assesses the length of time that yeast cells remain viable in a non-dividing state, and is considered to model the aging of quiescent cells in higher organisms [15]. Using this assay, studies have demonstrated interventions, genetic and otherwise, that regulate lifespan not only in yeast, but also in higher organisms, including mammals. For example, calorie restriction (CR) extends yeast chronological lifespan and has been shown to increase lifespan by up to 40% in mice, while impairment of the conserved insulin/IGF-1-like and TOR pathways produces similar gains in both organisms [7], [8], [16], [17]. The second model system, the replicative lifespan of mammalian cells in culture, reflects the propensity of cells to senesce *in vivo* due to the accumulation of genotoxic damage, as well as other types of cellular stress. Such cells accumulate with age in several tissues [18]–[21] and can contribute to age-related pathology [22]. Here we show that two manipulations (genetic and dietary) aimed at producing a methionine-restricted state robustly extend the chronological lifespan of yeast cells. During the initial preparation of this manuscript, Wu *et al*. also demonstrated that dietary Meth-R extends yeast chronological lifespan [23], consistent with our findings, but here we also describe several of the underlying mechanisms. Moreover, we also show that genetic Meth-R confers stress tolerance to both yeast and mammalian cells, engages the retrograde response, and dramatically extends the replicative lifespan of cultured mouse and human fibroblasts.

## Results

### Genetic methionine restriction extends yeast chronological lifespan

**Figure 1:**
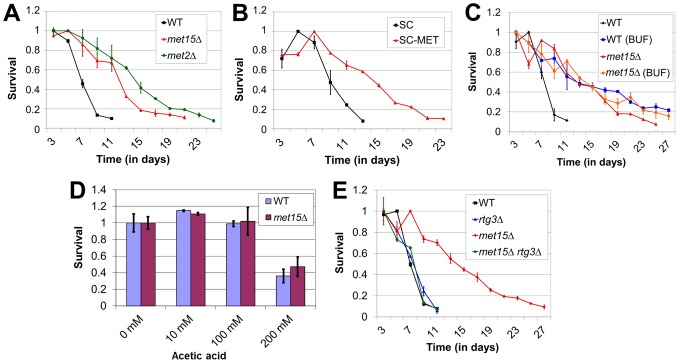
Extension of yeast chronological lifespan (CLS) by methionine restriction (Meth-R). (A) Effects of methionine biosynthetic deficiencies (genetic Meth-R) on CLS, (B) Effect of dietary Meth-R on CLS, (C) Effect of pH-buffering (BUF) on CLS, (D) Survival of wild-type and *met15Δ* cells following treatment with increasing amounts of acetic acid, (E) Requirement of the retrograde response for CLS extension by genetic Meth-R. For all panels, bars denote standard error of the mean (SEM).

To test the possibility that lifespan extension by Meth-R could be modeled in a highly genetically tractable organism, we made use of the yeast chronological aging assay, which measures the length of time that yeast cells remain viable in a non-dividing state. As mentioned above, a recent study has demonstrated that dietary Meth-R robustly extends yeast chronological lifespan [23]. Our aim, however, was to develop cell-based model systems wherein the methionine-restricted state is produced by genetic means, which has the benefit of allowing all aging experiments to be performed using a single media preparation. Although the intent in preparing matched normal and methionine-limited media is to hold the concentrations of all other components constant, in practice, small differences between media preparations may somewhat confound interpretation of studies utilizing dietary Meth-R. To test whether genetic interventions that abrogate methionine biosynthesis extend lifespan in yeast, we assessed the chronological lifespans of yeast deleted for either of two genes involved in methionine production (*MET2* and *MET15*). We found these mutants to be significantly longer-lived than wild-type (p<0.0001) (Fig. 1A). To determine the extent to which this intervention (“genetic Meth-R”, as we term it) recapitulates dietary Meth-R in our strain background, we measured the survival of wild-type haploid yeast cells aged in either normal media or media lacking both methionine and cysteine, the latter of which can be converted into methionine *via* a salvage pathway. We found that cells grown in methionine-restricted media showed a robust extension of lifespan (p<0.0001), to a similar extent as observed for genetic Meth-R (Fig. 1A–B). This suggests that genetic Meth-R is at least as efficient as dietary methionine limitation in producing the methionine-restricted state. For subsequent experiments characterizing genetic Meth-R in yeast, we chose to use the *met15Δ* deletion (as opposed to *met2Δ*), owing to the fact that cells of the haploid BY4741 Yeast Knockout Collection already lack *MET15*, thus facilitating study of the effects of additional mutations on lifespan extension by *MET15* deletion.

While we found that Meth-R in yeast recapitulated the benefits of this manipulation to rodents, it was unclear whether the benefit to yeast was due to methionine limitation, specifically, or merely the consequence of reducing the cellular pool of any single amino acid. It was previously demonstrated that limitation of total amino acids extends yeast chronological lifespan, and a subsequent study revealed that removal of either asparagine or glutamate from culture media results in a moderate extension of median lifespan [24], [25]. The latter finding, however, is difficult to reconcile with data from another group indicating that cells aged in media containing 20-fold higher levels of glutamate than normal are also long-lived [23]. What is clear, however, is that amino acid availability can have profound consequences for the stationary phase survival of yeast. To determine whether the simple removal of any one amino acid was sufficient to extend chronological lifespan, we aged wild-type yeast in normal media, as well as four other media formulations, each lacking a randomly selected amino acid (lysine, valine, isoleucine or threonine). We observed no lifespan extension for cells aged under these conditions (Fig. S1A). In addition, we found that genetic restriction of lysine, through deletion of the *LYS2* gene, was similarly incapable of extending chronological lifespan (Fig. S1B). These data therefore indicate that the mere limitation of any particular amino acid is insufficent to extend chronological lifespan. Rather, the Meth-R-responsive pathway(s) that confer extended lifespan in yeast do so in response to manipulations that specifically restrict methionine.

### Improved longevity of methionine-restricted yeast is due to reduced acid accumulation

Cells undergoing chronological aging are subjected to multiple stresses, including nutrient-limited conditions and the accumulation of acetic acid. In fact, acetic acid toxicity has been reported to be the major cause of death during yeast chronological aging, and dietary and genetic interventions that extend chronological lifespan result in reduced acetic acid accumulation and/or improved acid tolerance as compared with controls [26]. In addition, it was demonstrated that high acetate levels do not confer toxicity at a neutral pH. To test whether the lifespan extension of methionine-restricted yeast might be due to altered acid metabolism, we determined the lifespans of cells grown in media pH-buffered using 2-(*N*-morpholino)ethanesulfonic acid (MES, pH 6.0). Under such conditions, aging cells are incapable of acidifying the culture medium below a pH of 5.5, and acetate remains in its conjugate base form, which is non-toxic. In MES-buffered media, the lifespans of wild-type cultures were extended to that of *met15Δ* (Fig. 1C), with no significant difference in overall longevity (p = 0.5598). Similar results were observed by Wu *et al*. when assessing the effect of pH buffering on the lifespan of yeast chemically restricted for methionine [23]. Therefore, the benefit of Meth-R to yeast chronological lifespan is either reduced acid accumulation, resistance to acetic acid-induced death, or some combination thereof. To discriminate between these possibilities, we tested whether acid accumulation was affected in methionine-restricted cultures by measuring the pH of normally aged cultures (i.e., unbuffered) at varying intervals. We found that there was a direct correlation between age-related pH and lifespan, with cultures of long-lived cells genetically restricted for methionine (*met15Δ*) demonstrating higher pH values. Measurements of WT cultures revealed a pH of 3.5 after 13 days of aging, whereas *met15Δ* cultures were less acidic even 12 days later (Day 25, pH 3.75). While this finding might superficially appear to be contrary to a previous report that pH is not altered between normal and methionine-restricted yeast during chronological aging [23], the authors of the aforementioned study assessed pH only at one timepoint, after 2 days of aging, and were therefore unable to report on subsequent pH changes. To determine whether genetic Meth-R might also render cells more tolerant to acetic acid-induced death, we assessed the survival of both wild-type and *met15Δ* cells after treatment with an extrinsic acetic acid source at a concentration similar to that typically achieved during chronological aging (10 mM), as well as at higher concentrations (100–200 mM), in order to offset the transient nature of the treatment (acetic acid represents a persistent or chronic stress during chronological aging). All cells were similarly sensitive to acetic acid-induced death, regardless of genotype (Fig. 1D), indicating that genetic Meth-R does not also confer acetic acid tolerance. Our data are therefore consistent with genetic Meth-R extending yeast chronological lifespan primarily by reducing acid accumulation.

### Extension of yeast chronological lifespan by methionine restriction requires the retrograde response

As methionine-restricted growth resulted in reduced acidification of yeast culture media, we hypothesized that Meth-R might alter the expression of factors involved in cellular metabolism. Because the so-called retrograde response pathway regulates nuclear gene expression in response to nutritional stress and mitochondrial dysfunction [27], [28], we considered the possibility that this pathway might be upregulated in methionine-restricted cells. The key mediator of retrograde signaling is the translocation of the Rtg1/3 transcription factor complex to the nucleus, where it alters expression of a number of genes, enriched for factors involved in metabolism, chromatin remodeling and genome stability. Activation of retrograde signaling has been shown to extend the replicative lifespan of yeast mother cells [29]. Furthermore, it is known that TOR signaling inhibits the retrograde response, and that inhibition of TOR extends both replicative and chronological lifespan [30]–[32]. To explore putative connections between Meth-R and the retrograde response, we asked whether the altered transcriptional program of methionine-restricted cells required *RTG3* (which is indispensible for retrograde signaling in yeast [27], [28]). Towards this end, we performed expression profiling of aged (Day 3) wild-type, *met15Δ*, and *met15Δ rtg3Δ* cells. We chose the Day 3 timepoint as it corresponds approximately to the timing of the diauxic shift, when important changes in the metabolic program take place, and hypothesized that the modulation of such changes may underlie the decreased acid accumulation and extended lifespan of *met15Δ* cells. Of 1625 probes revealing differential gene expression in *met15Δ* cells as compared with wild-type, the altered expression of 313 (19%) was either blunted or absent in *met15Δ rtg3Δ* cells lacking a functional retrograde response (Tables 1 and S1). Using the DAVID functional annotation tool, [33], we found that 222 functional categories were enriched within the identified probe sets (88 for upregulated genes, 134 for downregulated genes; Table S2). Furthermore, several of the enriched categories (particularly those associated with upregulated genes) correspond to gene groupings with functions in metabolism, e.g., sulfur metabolic process (p = 0.00011), acetyl-CoA metabolic process (p = 0.00792), tricarboxylic acid cycle (p = 0.00328), glycolysis/gluconeogenesis (p = 0.03555), and generation of precursor metabolites and energy (p = 0.02101) (see Table S2 for complete list). Differentially-expressed genes were also enriched for factors involved in protein turnover (p = 0.01162) and proteasome function (p = 0.04398), which is intriguing given that protein quality control mechanisms have been implicated in the regulation of longevity [34]. Consistent with our model of Meth-R-mediated yeast lifespan extension, it is likely that perturbation of one or more of these processes contributes to the reduced acid accumulation and extended chronological lifespan of methionine-restricted cells.

**Table 1:** List of genes differentially-expressed in aged (Day 3) yeast by genetic Meth-R, dependent on the retrograde response.

**ABF1**	**HOM6**	**RTC2**	**YIM1**	EXG2	NMA1	URA1
**ACO2**	**HSP32**	**SDT1**	**YIR042C**	EXO1	OPT2	YAR028W
**ACS1**	**HSP33**	**SEO1**	**YJL132W**	FCY1	OST4	YAR029W
**ADP1**	**SNO4**	**SHH3**	**YKL069W**	FCY21	PAM18	YBR016W
**ADY2**	**HUL4**	**SHH4**	**YLR143W**	FCY22	PIN3	YCL021W-A
**AIM19**	**ICS2**	**SIR3**	**YLR154W-E**	FDH1	PIS1	YCR043C
**ALO1**	**ILM1**	**SNF1**	**YLR241W**	FRE3	PLB3	YDL012C
**APL3**	**IML1**	**SNO2**	**YLR346C**	FRT1	PMA1	YDR210W
**AQY1**	**INP54**	**SNO3**	**YMR084W**	GAS1	PMU1	YDR222W
**ARG3**	**KAP123**	**SNT309**	**YNL146W**	GAS2	PRM10	YER163C
**ARG5,6**	**KEL1**	**SNZ2**	**YPL109C**	GAS3	PTP2	YGR035C
**ARO10**	**KNH1**	**SNZ3**	**YPL113C**	GAS4	PUF3	YGR079W
**BDS1**	**KXD1**	**SPE3**	**ZPS1**	GIC2	PUG1	YIP3
**BLM10**	**LYS9**	**SPL2**	**ZRG17**	GND1	QDR2	YIR018C-A
**BOP2**	**MCH2**	**SPO14**	ADH6	HAC1	RAD51	YJL171C
**COG1**	**MCX1**	**SPO19**	ADK2	HBN1	RFS1	YLR040C
**COG8**	**MDS3**	**SPS100**	ALR2	HDA1	RFX1	YLR157W-E
**CRC1**	**MET5**	**STP4**	APJ1	HEM13	RGI1	YLR159W
**CRF1**	**NCA2**	**SUL1**	ARE2	HMLALPHA2	RGS2	YLR177W
**CSI1**	**NDE2**	**TAT1**	ASK1	HMRA2	RIM8	YLR194C
**DER1**	**NTA1**	**TCB1**	ASP3-1	MATALPHA2	RNR1	YLR342W-A
**DFR1**	**NUT1**	**THI4**	ASP3-2	HOG1	ROG3	YML119W
**DIS3**	**OPT1**	**TRR2**	ASP3-3	HXT4	ROX1	YMR122W-A
**DPP1**	**OSH2**	**UBA1**	ASP3-4	IMD4	RPS9A	YMR265C
**DUF1**	**PAU17**	**UBC12**	BTN2	IZH3	RSB1	YMR295C
**ECL1**	**PCL6**	**VBA3**	BUD23	KTI11	SCS7	YNL155W
**ECM1**	**PDC6**	**VBA5**	CAR1	LIP5	SCW10	YNL234W
**ECM4**	**PEP5**	**VPS13**	CDC3	LSB1	SFK1	YNR014W
**EMC2**	**PGK1**	**VPS15**	CHS1	MAL12	SFM1	YOL014W
**EMI5**	**PHO84**	**VPS30**	CIN5	MAL32	SMP1	YOL036W
**ENV11**	**PKP2**	**VPS33**	CIS3	MCA1	SOH1	YOR338W
**ERG24**	**PMA2**	**VPS55**	CLA4	MCM1	SPI1	YOR378W
**ERR1**	**PPH22**	**WTM1**	COA2	MDM30	SPT15	YOR389W
**ERR2**	**PRE1**	**XYL2**	COX23	MEP2	SSL2	YPC1
**ERR3**	**PRE3**	**YBL029W**	CPR6	MF(ALPHA)1	STE3	YPK2
**ESC8**	**PTC5**	**YBL039W-B**	CTR1	MGA1	STP3	YPL014W
**ETR1**	**PUT4**	**YBP1**	CYC7	MIC17	STS1	YPP1
**EUG1**	**QRI7**	**YBR285W**	DAL3	MID2	SUN4	YPR153W
**FMP27**	**RAS1**	**YBT1**	DED1	MIG2	TIM17	YPS3
**FMP45**	**RAV1**	**YDL218W**	DIF1	MIM2	TOS1	YPS7
**GPI13**	**RFC1**	**YFL042C**	DOG2	MMT1	TOS3	YSR3
**GPM2**	**RNR2**	**YGL242C**	DOT6	MND1	TPO4	YSY6
**GRE1**	**ROT2**	**YGR026W**	DUR3	MPH2	UBP9	
**GTO1**	**RPI1**	**YHR022C**	EHT1	MPS2	UGA4	
**HBT1**	**RPN7**	**YIL001W**	ERG4	MRS3	UPS2	
**HES1**	**RRI2**	**YIL029C**	ESA1	MVD1	UPS3	

Genes indicated in **bold** are upregulated; all other genes are downregulated. Underlining of adjacent gene names indicates cases where probes are incapable of discriminating between highly similar genes. For all probe set IDs for which gene names are unavailable, systematic names are given.

In order to directly test whether the extended lifespan of methionine-restricted yeast cells required the retrograde response, we assessed the chronological lifespan of *met15Δ* cells that also lacked *RTG3*. We found that lifespan extension by genetic Meth-R was indeed dependent upon *RTG3* (Fig. 1E), as cultures of *met15Δ rtg3Δ* double mutant cells failed to persist longer than wild-type (p = 0.4405). Furthermore, the impairment of genetic Meth-R-dependent lifespan extension in cells lacking the retrograde response was not due merely to non-specific sickness, as we found that *rtg3Δ* single mutant cells were not shorter-lived than wild-type. Collectively, these experiments have revealed an integral role for retrograde signaling in the extension of yeast chronological lifespan by genetic Meth-R.

### Altered tRNA metabolism and extension of yeast chronological lifespan

**Figure 2:**
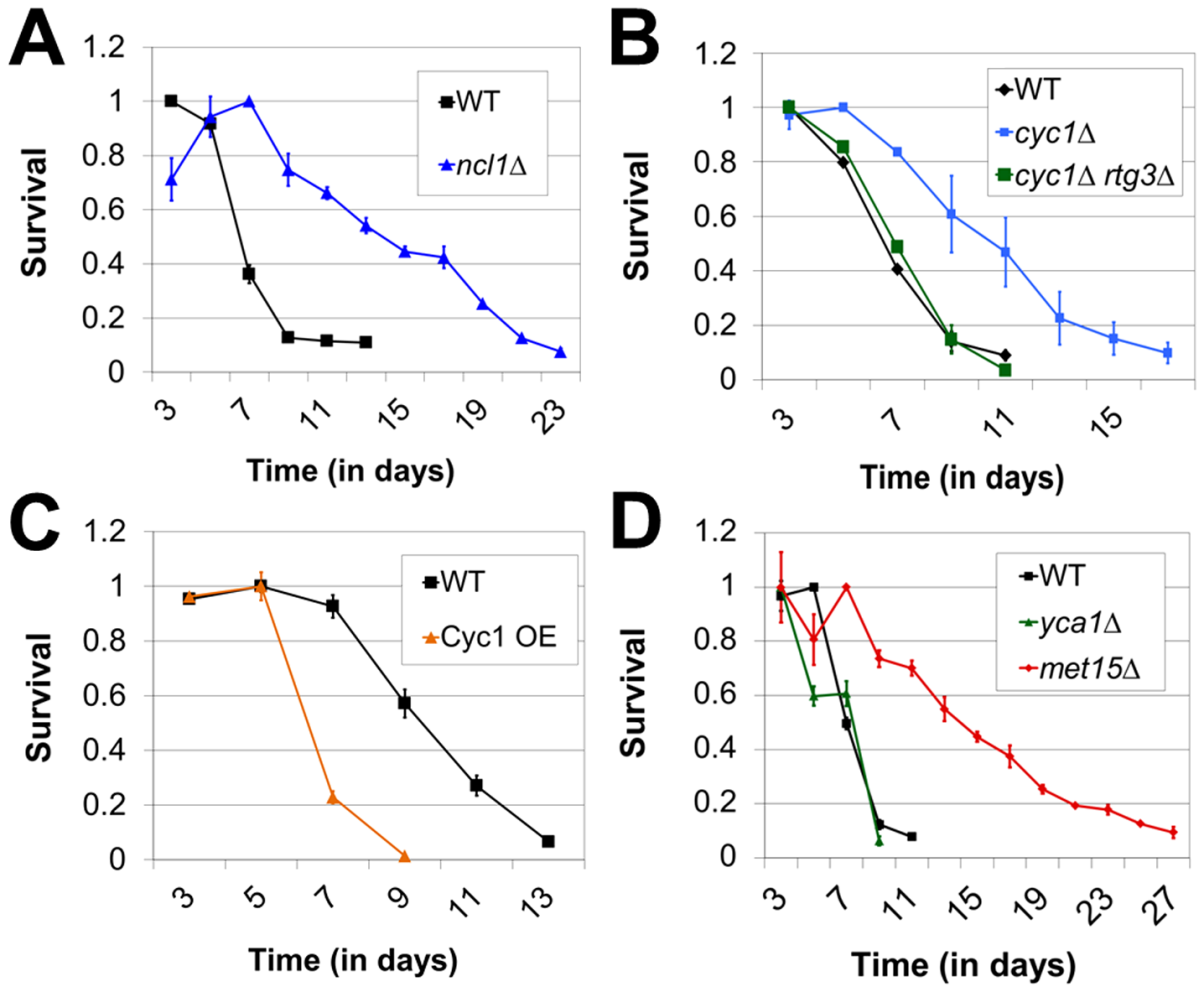
Extension of yeast CLS by mutations predicted to phenocopy the cellular consequences of Meth-R. (A) Effect of tRNA hypomethylation on CLS, (B) Effect of cytochrome C deficiency on CLS, dependent on the retrograde response, (C) Effect of cytochrome C overexpression on CLS, as compared with control cells (vector only), (D) Deletion of yeast metacaspase does not extend CLS. For all panels, bars denote SEM.

Studies have demonstrated that growth in media lacking methionine results in hypomethylation of tRNAs [35], [36], raising the possibility that the extension of yeast lifespan observed upon Meth-R is mediated by altered tRNA metabolism. Prompted by this, as well as a report that cells lacking the Trm9 tRNA methyltransferase show extended longevity in the chronological aging assay [37], we determined the chronological lifespans of several yeast strains, each lacking one of several different tRNA methyltransferase gene (not shown). Of these, only cells lacking the *NCL1* gene were found to be long-lived, showing a dramatic increase in lifespan as compared with wild-type (p<0.0001) (Fig. 2A). Notably, Ncl1 is responsible for all m5c methylation of tRNAs [38], [39]. Similar to the results for Meth-R, the lifespan of long-lived *ncl1Δ* cells was inversely proportional to the extent to which they acidified their culture media (Day 23, pH 3.75 and Day 13, pH 3.5; *ncl1Δ* and WT, respectively). Additionally, the buffering of media (MES, pH 6.0) used for aging of wild-type and *ncl1Δ* cells removed the benefit of *NCL1* deletion to the relative longevity of these cells, rendering all cells long-lived (not shown). To further explore connections between Meth-R and tRNA metabolism, we used qRT-PCR to determine the relative abundance of three randomly selected tRNA species (tRNA-elongator-Met, tRNA-Cys, and tRNA-Ser) in aged wild-type cells, *ncl1Δ* cells and cells restricted for methionine. As a positive control for the sensitivity of our assay, we were able to detect elevated tRNA levels in cells lacking the Rny1 ribonuclease, which cleaves tRNAs under conditions of stress. Importantly, we found that all long-lived cells showed elevated accumulation of the interrogated tRNAs as compared with wild-type (∼2-fold for each tRNA in methionine-restricted or *ncl1Δ* cells; p = 0.0085–0.0339) (Fig. S2). These studies therefore suggest that tRNA metabolism may indeed play an important role in propagating the effects of Meth-R.

### Evidence for involvement of cytochrome C in the methionine restriction–induced activation of the retrograde response

Data from two published studies suggested to us a putative mechanism by which Meth-R might alter tRNA metabolism to promote longevity. First, a recent study in mice has shown that tRNA binds to cytochrome C and inhibits its ability to activate caspase [40]. Second, activation of the retrograde response in yeast is induced by mitochondrial dysfunction, nutritional stress and defects in metabolism [27], [28]. As cytochrome C supports oxidative phosphorylation and functions in mitochondria, we were intrigued by the possibility that elevated tRNA levels in methionine-restricted yeast might inhibit some aspect of cytochrome C function, thereby activating the retrograde response. We thus hypothesized that a reduction in the levels of cytochrome C, by deletion of a gene encoding one of its isoforms (*CYC1*), might mimic a partial inhibition of cytochrome C function, thereby activating the retrograde response and extending yeast chronological lifespan. As predicted, we found that *cyc1Δ* cells were long-lived (p = 0.0195) (Fig. 2B). In addition, and similar to the results for Meth-R, we found a requirement for retrograde signaling in extension of lifespan by loss of Cyc1, as *cyc1Δ rtg3Δ* cells were not longer-lived than wild-type (p = 0.538). To further explore the role of cytochrome C in regulating yeast chronological lifespan, we introduced into wild-type yeast an overexpression construct encoding cytochrome C. Analysis of the resulting strain revealed that forced expression of cytochrome C negatively impacted chronological lifespan as compared with a vector-only control (9 days vs. 13 days; p<0.0001) (Fig. 2C). To insure that the observed reduction in lifespan was not due simply to non-specific toxicity associated with elevated levels of cytochrome C, we confirmed that Cyc1 overexpression did not impair cell growth (not shown). These results are therefore consistent with a model wherein a threshold level of functional cytochrome C precludes activation of the retrograde response, this in turn having negative implications for cellular longevity.

While the requirement for the retrograde response, and extension of lifespan by reduced acid accumulation are sufficient to explain the increased longevity of methionine-restricted cells, impairment of cytochrome C is a putative step in this extension, and given the role of Cyc1 in yeast programmed cell death (PCD), we sought to determine whether a blunting of PCD might partially underlie the Meth-R phenotype. Therefore, we tested whether deletion of the yeast metacaspase (Yca1), a key mediator of acetic acid-induced PCD [41], [42], might phenocopy the extended longevity of Meth-R. Although Yca1-independent acetic acid-induced PCD pathways also exist in yeast, PCD caused by Cyc1 release requires Yca1 [43]. Similar to a previous study showing only a very modest improvement in chronological lifespan by deletion of *YCA1* [44], we saw no benefit of *yca1Δ* to yeast longevity (Fig. 2D), indicating that inhibition of Cyc1-dependent PCD does not contribute significantly to the extended lifespan of methionine-restricted yeast.

In total, however, our genetic studies exploring the mechanism(s) underlying Meth-R in yeast suggest that this intervention may extend yeast chronological lifespan, at least in part, by causing the accumulation of tRNAs, which, through effects on cytochrome C, alters nuclear gene expression and acid metabolism *via* the retrograde response.

### Methionine restriction of yeast confers resistance to multiple cellular stresses

**Figure 3:**
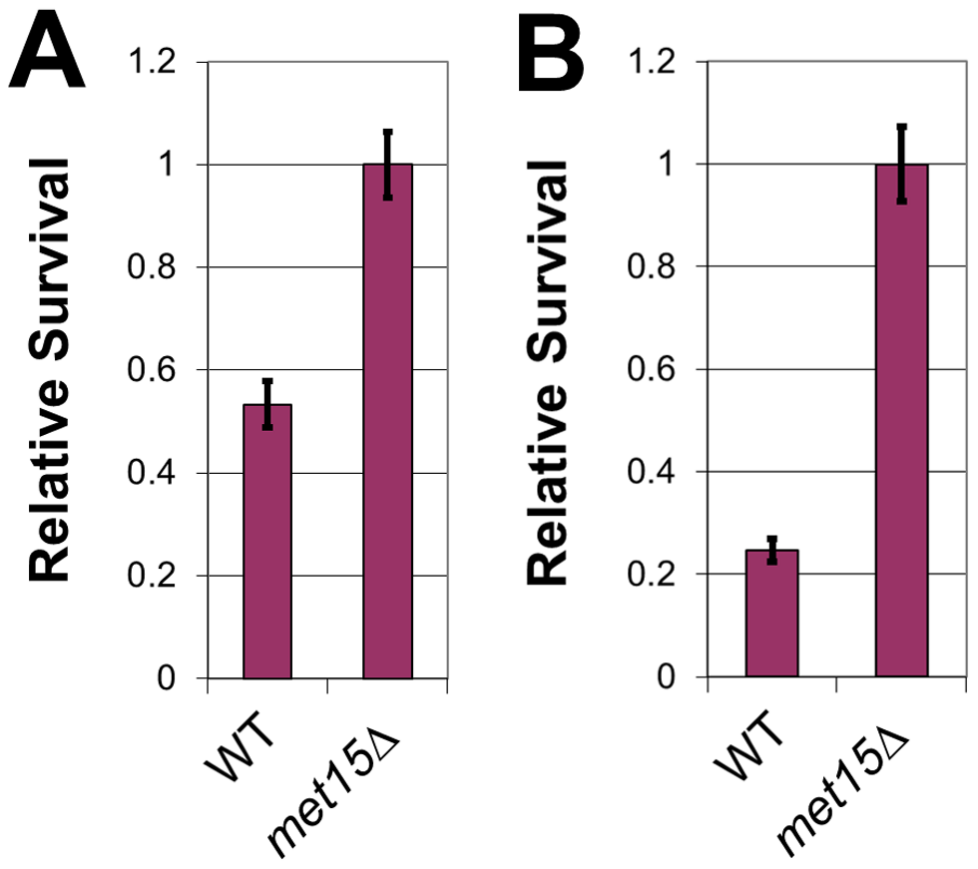
Stress tolerance of methionine-restricted yeast. (A) Relative survival (compared with cells grown at the permissive temperature) of yeast strains, as indicated, to 55°C heat shock, (B) Relative survival (compared with cells grown in normal, non-toxic medium) of cells incubated in medium containing the toxic compound 1,10-phenanthroline (10 mM). Values are normalized to survival of *met15Δ* cells. For both panels, bars denote SEM.

As the benefits of Meth-R may be conferred, at least partially, through stress-responsive retrograde signaling, we wondered whether improved stress tolerance might be associated with Meth-R-dependent longevity. Consistent with this idea, genetic Meth-R (*met15Δ*) was previously shown to confer resistance to oxidative (diamide) and heavy metal stresses (methyl mercury and cadmium) [45]–[47]. Further, as *met15Δ* cells, and those chemically restricted for methionine (Fig. 1A–B) [23], are long-lived in the chronological aging assay, these cells can thus be considered to be resistant to the nutritional stresses encountered during aging. These findings suggested to us that Meth-R might promote generalized resistance to cytotoxic insults. We therefore tested whether methionine-restricted yeast might also be resistant to heat stress. Specifically, we assessed the survival of wild-type and *met15Δ* cells subjected to heat shock at 55°C for 5 mins as compared with those incubated at the standard temperature of 30°C. Survival of *met15Δ* cells following heat shock was found to be nearly 2-fold greater than that of wild-type control cells (Fig. 3A).

1,10-phenanthroline chelates divalent metal cations and is highly toxic to yeast cells. To determine whether the apparent generalized stress tolerance conferred by Meth-R might also protect cells from the toxic effects of this agent, we incubated both wild-type and *met15Δ* cells at 30°C in SC medium containing 10 mM 1,10-phenanthroline and assessed their survival after 24 hrs. Cells undergoing genetic Meth-R demonstrated nearly 4-fold greater survival than wild-type cells (Fig. 3B). Therefore, in addition to oxidative damage and heavy metal stress, genetic Meth-R also protects against nutrient-limited conditions, temperature stress and 1,10-phenanthroline toxicity.

### Genetic restriction of methionine in cultured mammalian cells confers stress resistance, extends lifespan and alters the expression of NFκB signaling factors

**Figure 4:**
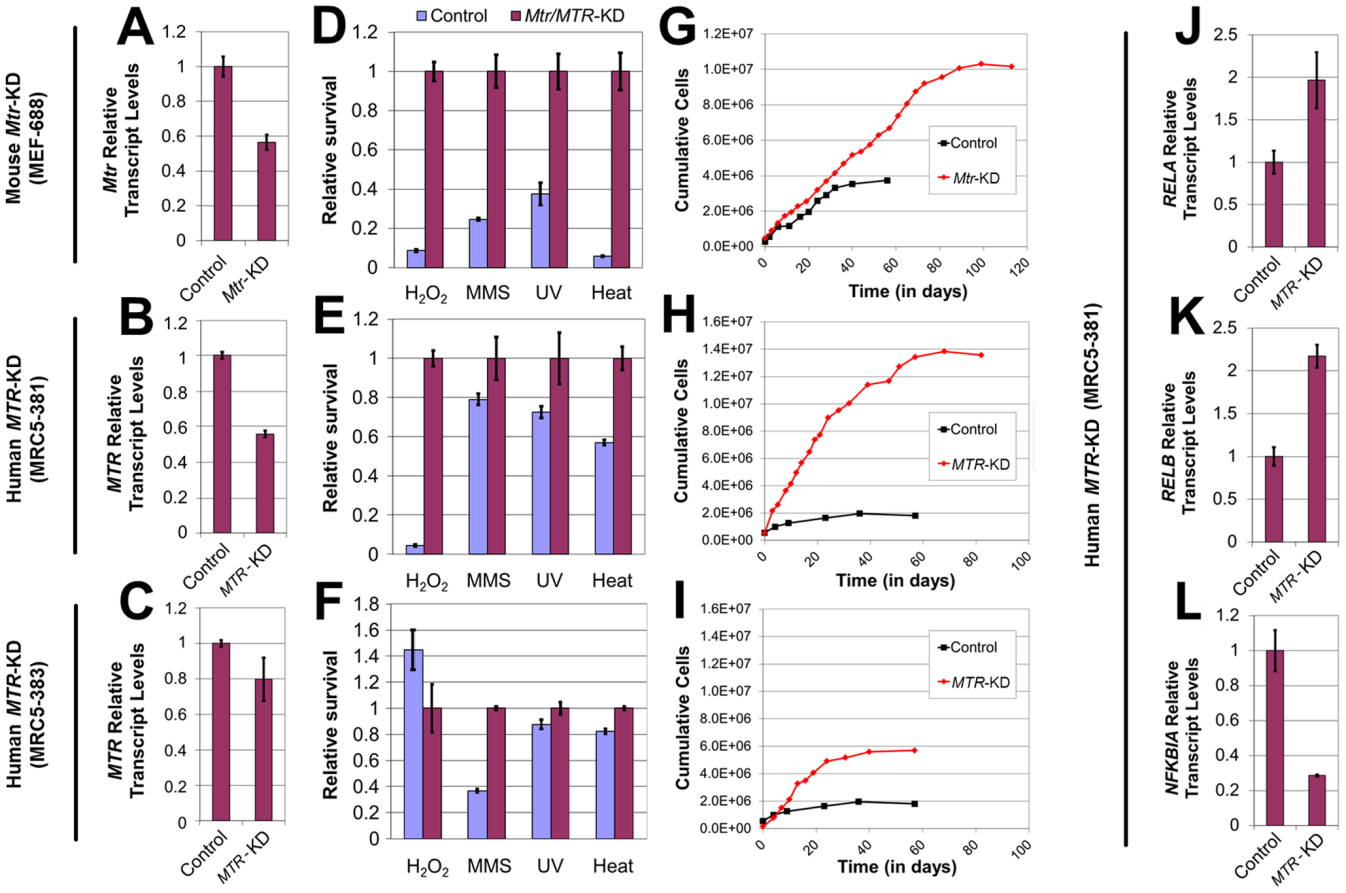
Effects of genetic Meth-R on stress tolerance, replicative lifespan and the expression of NFκB signaling genes in cultured mammalian cells. (A–C) qRT-PCR determination of the extent of methionine synthase (*Mtr/MTR*) transcript depletion in cultured (A) mouse embryonic fibroblasts (MEF-688), as well as (B) human MRC5-381 and (C) human MRC5-383 fibroblasts (C). Transcript levels are normalized to those in mock-infected control cells. (D–F) Relative post-stress survival of methionine synthase knockdown fibroblasts (purple) (D) MEF-688, (E) MRC5-381, and (F) MRC5-383, as compared with mock-infected controls (light blue) (values shown are computed relative to non-stressed cell viability, and normalized to survival of *Mtr/MTR-*KD strains). (G–I) Replicative lifespan analyses of methionine synthase knockdown strains (G) MEF-688, (H) MRC5-381, and (I) MRC5-383, as compared with mock-infected controls. (J–L) qRT-PCR determination of (J) *RELA,* (K) *RELB* and (L) *NFKBIA* transcript levels in *MTR-*knockdown human fibroblasts (MRC5-381), as compared with transcript levels in mock-infected control cells. For panels A–F and J–L, bars denote SEM.

Given that Meth-R conferred both stress resistance and lifespan extension to yeast cells, and has been shown to extend the healthspan and overall lifespan of rodents, we hypothesized that this intervention might promote the upregulation of cellular stress responses in cultured mouse cells, with a concomitant improvement in post-stress survival. To test this hypothesis, we constructed lentiviruses expressing shRNAs against transcripts encoded by murine *Mtr* or human *MTR* (methionine synthase), and used them to generate fibroblast strains wherein methionine synthase was depleted, i.e., genetic Meth-R. *Mtr* transcript depletion in MEFs (mouse embryonic fibroblasts; strain MEF-688) was confirmed by qRT-PCR, with *Mtr* knockdown (*Mtr*-KD) cells showing approximately half the abundance of control *Mtr* mRNA levels (Fig. 4A). The resultant *Mtr-*KD and mock-infected control MEFs were then subjected to a panel of stresses typically used to assess stress tolerance in mammalian cells, including: temperature stress, treatment with the genotoxic drug methyl methanesulphonate (MMS), irradiation with UV light, and oxidative stress *via* hydrogen peroxide treatment. Compared with controls, *Mtr*-KD MEFs showed improved survival following all stress exposures (Fig. 4D). We also used shRNAs to reduce *MTR* levels in human MRC5 fibroblasts, and assessed stress resistance in these cells. *MTR*-KD human cell strains showed a 44% (MRC5-381) and 20% (MRC5-383) reduction in *MTR* transcript levels, respectively (Fig. 4B, 4C). MRC5-381 cells were found to be resistant to all stress treatments, albeit to a lesser degree than observed for *Mtr-*KD mouse cells (Fig. 4E). Interestingly, MRC5-383 cells, with higher *MTR* transcript levels as compared with MRC5-381, showed a weaker stress tolerance phenotype, demonstrating resistance only to MMS treatment (Fig. 4F).

These results prompted us to test whether such improved stress tolerance might be associated with extended replicative lifespan in culture. We serially passaged the mock-infected and *MTR*-KD fibroblasts described above, until these cultures reached their respective replicative lifespan endpoints and were unable to further proliferate. *Mtr*-KD MEFs showed both an overall reduced doubling time and a marked improvement in replicative capacity (44 PD, *Mtr-*KD; 34 PD, control) (Fig. 4G). Given that *Mtr* knockdown was initiated at PD11, this represents a lifespan extension of 43%, as measured from the start of genetic Meth-R. Using a more stringent analysis comparing overall lifespans irrespective of the timing of Meth-R initiation, the increase in maximum population doubling levels was 29%. By either analysis, it is clear that genetic Meth-R efficiently extended the replicative lifespan of cultured MEFs. Similar levels of *MTR* depletion (∼44%) in human MRC5-381 fibroblasts also accelerated their growth rate and extended their maximal lifespan (70 PD, *MTR-*KD; 58 PD, control) (Fig. 4H). As genetic Meth-R was initiated at PD46 in human cells, this represents a lifespan extension of 100%, or 21% when comparing overall lifespans. Interestingly, more moderate depletion of *MTR* transcripts in MRC5-383 cells also resulted in an accelerated growth rate and extension of replicative lifespan (65 PD, *MTR-*KD; 58 PD, control) (Fig. 4I), but not to the same extent as observed for MRC-381 fibroblasts. This intermediate extension of lifespan (63% from the start of Meth-R, 12% overall), in combination with the observation of an attenuated stress resistance phenotype for MRC5-383 cells, suggests that there is a dose-dependency of methionine synthase for inhibition of pathways underlying the benefits of genetic Meth-R. Furthermore, the fact that the efficiency of *MTR* depletion is directly correlated with the robustness of the resulting stress tolerance and lifespan extension phenotypes confirms that these benefits are not the result of putative technical artifacts associated with strain construction by lenitiviral infection, but rather, that they are specifically engendered by reduced methionine synthase levels. In addition, we note that extension of proliferative lifespan was not caused by slower cell division, because Mtr/MTR knockdown actually increased cell division rates and yielded a greater total number of cell divisions.

Regarding the mechanism by which genetic Meth-R confers lifespan extension to mammalian cells, we considered the possibility that, similar to the case in yeast (Fig. 1E), retrograde signaling might be involved in the Meth-R-dependent extension of replicative lifespan that we observed for mammalian cells. In mammals, multiple retrograde signaling mechanisms exist that are functionally similar to the yeast RTG system in that they alter nuclear gene expression in response to mitochondrial dysfunction, stress and other cues [27], [28]. For example, NFκB signaling is activated by mitochondrial stress [48], [49]. NFκB activity is regulated by multiple mechanisms, including altered transcription of NFκB components [50], [51]. To address the possibility that the NFκB pathway is engaged by genetic Meth-R, we used qRT-PCR to assess relative expression levels, in human *MTR-*KD and control cells, of transcripts encoding three factors involved in NFκB signaling (*RELA*, *RELB* and *NFKBIA*). Our analyses demonstrated that the levels of transcripts encoding the NFκB family members RelA and RelB were 2-fold higher in long-lived *MTR-*KD cells (MRC5-381), whereas *NFKBIA*, which encodes the NFκB inhibitor IκB, was downregulated nearly 4-fold (Fig. 4J-L). Together, these results suggest that NFκB signaling is activated by genetic Meth-R, and further, support the hypothesis that Meth-R promotes the stress tolerance and extended replicative lifespan of mammalian fibroblasts through NFκB-dependent changes in gene expression.

## Discussion

Although it has been suggested that increased cellular stress resistance contributes to the extended lifespan imparted by longevity-promoting manipulations, few studies provide evidence for such a mechanism in the case of Meth-R. A key study characterizing Meth-R in mice showed that a methionine-restricted diet improves the resistance of hepatocytes to oxidative stress injury by acetaminophen injection *in vivo* [3], while a more recent report found that methionine-restricted rats have reduced blood levels of oxidative stress biomarkers [5]. Consistent with these findings, a third group reported that mitochondrial ROS are decreased in methionine-restricted rats, along with oxidative damage to mtDNA [52]. It remains unclear, however, whether such effects are due to an increased resistance to oxidative stress, *per se*, or simply the generation of lower basal levels of ROS in methionine-restricted animals. To explore in detail the mechanisms connecting Meth-R, cellular stress resistance and longevity, we developed two novel genetic systems using yeast and mammalian cells. Utilizing these, we have demonstrated, for the first time, that genetic Meth-R significantly extends the chronological lifespan of yeast, primarily by eliciting changes in metabolism that decrease acid accumulation. Notably, we found that Meth-R-dependent lifespan extension is equally robust whether engaged by dietary Meth-R or impairment of the cell's intrinsic ability to synthesize methionine. We have also found that Meth-R-dependent chronological lifespan extension is associated with improved resistance of yeast to cytotoxic stresses, including heat stress and 1,10-phenanthroline toxicity. These are in addition to the increased resistance of methionine-restricted yeast to oxidative and heavy metal stresses, as demonstrated previously [45]–[47]. Exploiting the utility of this system, we explored the genetic requirements for lifespan extension as conferred by genetic Meth-R, and found that that this benefit requires a functional retrograde response. Consistent with our findings (and during the preparation of our manuscript), growth in raffinose was reported to confer protection *via* the retrograde response against acetic acid-induced yeast cell death [53]. We also discovered that a large proportion of genetic Meth-R-induced transcriptional changes require that retrograde signaling remain intact, and further, that such changes are enriched for factors with roles in metabolism and other cellular processes implicated in the control of longevity (e.g., protein quality control and autophagy). For example, a gene encoding spermidine synthase (*SPE3*) was revealed by our analyses to be upregulated by Meth-R in a retrograde signaling-dependent fashion (Table 1). Spermidine, a natural polyamine, has been shown to extend the lifespan of yeast, flies, worms and human immune cells through activation of autophagy [54]. While we consider it likely that some of the identified expression changes underlie the stress tolerance, and reduced acid accumulation (and thus, extended lifespan) conferred by Meth-R, future studies will be necessary to determine precisely which factors are the key mediators of these phenotypes. In the current study, however, we have been able to make initial exploratory steps towards an elucidation of the mechanisms supporting this intervention. Our data are consistent with a model whereby methionine limitation results in hypomethylation and accumulation of tRNAs, which bind to cytochrome C, thereby activating the retrograde response. Retrograde signaling then promotes altered transcription of genes that regulate stress resistance, acid metabolism, and therefore, the chronological lifespan of yeast.

With respect to studies exploring the benefits of Meth-R to mammals, it has been demonstrated that certain tissues of methionine-restricted mice demonstrate stress tolerance *in vivo* [3]. However, subsequent studies performed by the same group revealed no stress resistance phenotypes in skin-derived fibroblasts harvested from methionine-restricted animals and cultured *ex vivo* under methionine-replete conditions [55]. Therefore, prior to our study, the question of whether individual mammalian cells might be stress resistant upon Meth-R had not been fully addressed. Using shRNA-mediated depletion of methionine synthase, we performed genetic Meth-R of cultured mouse fibroblasts and found that this intervention confers resistance to four distinct stresses (i.e., oxidative, genotoxic, heat and UV). Interestingly, this finding is reminiscent of results from two studies assessing the *ex vivo* stress resistance of cells from long-lived dwarf mouse strains [13], [14]. In these reports, fibroblasts harvested from Ames and Snell dwarf mice were shown to be substantially more stress tolerant than cells from control mice with typical lifespans. While the authors suspect that this phenotype might be due to altered insulin/IGF-1 signaling in these animals, we are intrigued by a recent report indicating that the cells of dwarf mice might have reduced methionine transport [56]. Beyond stress resistance, we demonstrate, for the first time, that Meth-R both reduces doubling time and extends the replicative lifespan of cultured MEFs. Meth-R-dependent extension of cellular lifespan *in vivo* likely has significant implications for the rate of mouse aging, especially given a recent study showing that selective clearance of p16(Ink4a)-positive senescent cells rescues age-related pathologies in a progeroid mouse model [22]. Just as intriguing is a recent study that revealed that rapamycin treatment, which extends mouse lifespan, reduces the incidence of senescent cells *in vivo* [57]. Our findings provide strong support for a causal relationship between Meth-R-induced stress resistance, extended replicative lifespan and improvements in rodent healthspan. Perhaps our most important finding, however, is that genetic Meth-R confers stress resistance to cultured human fibroblasts, as well as a reduced doubling time and an extension of replicative lifespan. In fact, the current study represents the first investigation into the putative effects of Meth-R on human cells for the purpose of ameliorating age-related phenotypes. Notably, our discovery of the beneficial effects of Meth-R on human cell replicative lifespan is supported by a recent study demonstrating that depletion of cystathionine beta synthase, a manipulation predicted to increase intracellular methionine levels, reduces the lifespan of cultured human endothelial cells [58]. Given the low methionine content of the vegan diet [59], dietary Meth-R is a conceivable strategy for promoting healthy aging in humans. However, it might not be practical or desirable due to potential side effects. Yet, and moreover, understanding Meth-R-responsive mechanisms promises to reveal key biochemical pathways impacting aging, which could be targeted by new strategies to ameliorate age-related diseases in an optimal fashion. While it is not currently known whether Meth-R is effective in humans, our discovery that it extends the replicative lifespan of cultured mouse and human cells strongly supports this possibility. With respect to the mechanistic basis of Meth-R, our findings suggest that the benefits conferred to human cells by this intervention are associated with activation of NFκB-mediated retrograde signaling. We anticipate that future work will ultimately identify which pathways engaged by Meth-R-dependent NFκB signaling underlie the pro-survival benefits of this intervention. Moreover, we are hopeful that Meth-R-dependent pathways might be selectively manipulated for the purpose of promoting healthy aging in humans, an effort that will be facilitated by our development of experimentally tractable Meth-R model systems.

## Materials and Methods

### Yeast strain and plasmid construction

All experiments were performed with haploid strains derived from the BY4741/BY4742 background (*his3Δ1, leu2Δ0, ura3Δ0*) [60]. Deletions of genes of interest were marked by the *KanMX* drug resistance cassette (with the exception of *met15Δ0* and *lys2Δ0*) and gene disruption was confirmed, as necessary, by PCR.

The yeast *CYC1* overexpression plasmid was constructed using a derivative of an Advanced Gateway Destination Vector [61]. The SacI/NotI fragment of plasmid pAG425-GPD-ccdB-EGFP, containing the *GPD* promoter, was replaced by a SacI/NotI fragment containing the *NOP1* promoter, in order to effect constitutive expression of genes of interest during stationary phase. The *CYC1* ORF (YJR048W) was introduced into the resulting Destination Vector through an LR recombination reaction using a pDONR221-derived Entry Clone, giving rise to a plasmid encoding *CYC1* (not fused to the *EGFP* gene), under control of *P_NOP1_*.

### Yeast chronological aging assays

Chronological aging assays were performed essentially as described (low aeration conditions) [62], but with some modifications. Cells were struck onto YPAD solid media from frozen stocks or dissection plates, and allowed to grow at 30°C for 48 hrs before colonies were inoculated into liquid synthetic complete (SC) medium. After an additional 48 hrs of growth, aliquots were transferred into fresh SC medium at a concentration of ∼2×10^5^ cells/ml and grown at 30°C. After three days of growth, post-diauxic shift, aliquots were removed at 48 hr intervals and their colony forming potentials assessed on YPAD agar plates (n = 4 for each condition). The point at which the percent survival for each culture, as compared with the Day 3 timepoint, was found to be less than 10% was considered the end of lifespan. This cut-off was chosen so as to avoid the potential confound of the GASP (Growth Advantage in Stationary Phase) phenotype, which is marked by the cyclical growth and death of a small population of cells [15].

Aging assays were performed using SC medium formulated as follows: 0.67% yeast nitrogen base without amino acids, 2% glucose, 0.45% casamino acids, 0.01% tryptophan, 0.008% adenine sulfate, and 0.009% uridine. For those experiments utilizing media lacking amino acids, the control media was formulated as follows: 0.67% yeast nitrogen base without amino acids, 2% glucose, 0.0018% adenine sulfate, 0.037% leucine and 0.0073%, each, of the following: alanine, arginine, asparagine, aspartic acid, cysteine, glutamine, glutamic acid, glycine, histidine, inositol, isoleucine, lysine, methionine, *para-*aminobenzoic acid, phenylalanine, proline, serine, threonine, tryptophan, tyrosine, uracil and valine. Amino acid-restricted media was prepared as described, but lacking the appropriate amino acids (i.e., lysine, valine, isoleucine, threonine, or in the case of methionine-restricted media, methionine and cysteine).

To assess the significance of lifespan differences between strains, 10% survival values (in days) were computed, and used to perform unpaired two-tailed t-tests. The statistical program Prism (GraphPad Software; La Jolla, CA) was used for these analyses.

### Yeast gene expression profiling

Total RNA was harvested from cultures of the indicated genotypes that had been aged three days, as described above. Following centrifugation, cells were mechanically disrupted using glass beads in combination with a Mini-BeadBeater 16 cell disruptor (Biospec; Bartlesvile, OK), total RNA was purified using the RNeasy Mini kit (Qiagen; Valencia, CA, USA) and subjected to genome-wide expression profiling using GeneChip Yeast Genome 2.0 arrays (Affymetrix; Santa Clara, CA, USA). Arrays hybridized with labeled cDNA probes were scanned and raw intensity values were background-corrected and RMA-transformed (Robust Multi-array Average; RMAExpress 1.0.4) to produce natural scale expression sets, which were then ranked in order of evidence for differential expression (compared with wild-type) with a fold-change cut-off of 2.0 (). Because these studies represented a preliminary exploration of the altered transcriptional profile of methionine-restricted yeast, we performed a single replicate for each condition, and were thus unable to compute p-values for expression data. Instead, our selection of a fold-change cut-off was informed by our previous experiences utilizing Yeast Genome 2.0 arrays to assess transcript levels in BY4741/BY4742 cells, where we found that fold-changes greater than 1.3 equated to p<0.05, as determined by the non-parametric statistical test, Rank Product [63]. In the current study, using a more stringent cut-off (2-fold), we identified genes that were differentially-expressed in *met15Δ* cells as compared with wild-type, but whose altered expression was dependent on the retrograde response (i.e., expression changes were reduced by at least 2-fold in *met15Δ rtg3Δ* cells as compared with *met15Δ* cells). To determine the enrichment of functional annotation categories comprising genes differentially-regulated by the retrograde response upon Meth-R (Table S2), we used the DAVID Bioinformatics Resource 6.7 functional annotation tool [33]. The complete, unprocessed dataset is available on the Gene Expression Omnibus (GEO) database (http://www.ncbi.nlm.nih.gov/geo/).

### Yeast stress and acetic acid resistance assays

Yeast temperature stress resistance assays were performed by subjecting non-dividing yeast (grown 48 hrs at 30°C in liquid SC medium) to incubation at either 30°C or 55°C for 5 minutes, before plating aliquots onto YPAD plates to allow for outgrowth of surviving cells. Similarly, 1,10-phenanthroline resistance was assessed by incubating non-dividing cells in liquid SC medium containing 10 mM 1,10-phenanthroline for 24 hrs at 30°C. Aliquots were then plated onto YPAD plates and grown for an additional 48 hrs at 30°C in order to determine the proportion of cells capable of forming colonies following cytotoxic exposure. For each set of experiments, n = 4 for each condition.

Acetic acid resistance assays were performed by treating yeast with increasing amounts of glacial acetic acid and assessing their survival post-treatment. Liquid cultures of yeast in SC medium were allowed to grow for 72 hrs at 30°C, at which point acetic acid was added to achieve final concentrations of 10 mM, 100 mM and 200 mM, respectively. Cultures were then grown at 30°C for an additional 210 mins, and aliquots removed and assessed for their relative colony forming potentials on YPAD plates (n = 4 for each condition).

### Ethics statement

Mice used for harvesting of embryonic fibroblasts (see below) were handled according to the relevant national and international guidelines, under protocol # 804470, approved by the Institutional Animal Care and Use Committee (IACUC) of the University of Pennsylvania Office of Regulatory Affairs. The IACUC office approved the animal use techniques employed in this study.

### Mammalian cell strain construction and maintenance

We generated lentiviruses expressing shRNAs against transcripts encoded by *MTR* (the mammalian homologue of the yeast *MET15* methionine synthase gene) to construct cell strains wherein methionine synthase is depleted. The shRNAs used were obtained from The RNAi Consortium shRNA library and are as follows: Human anti-*MTR*, TRCN0000035381; Mouse anti-*MTR*, TRCN0000251688. Lentiviruses encoding either these shRNAs or, as a control, a shRNA targeting luciferase, were used to infect non-immortalized mouse embryonic fibroblasts and human MRC5 fibroblasts at PD11 and PD46, respectively. Mouse embryonic fibroblasts were generated from Day 14 embryos of mouse strain C57Bl/6, whereas human MRC5 fibroblasts were obtained commercially (Coriell; Camden, NJ, USA). Parental fibroblast cell strains were cultured in DMEM supplemented with 15% fetal bovine serum (FBS), 1x penicillin/streptomycin, and 1x fungizone in 6% CO_2_ at 37°C. Unless otherwise indicated, mouse cells were incubated in ambient O_2_ and human cells were incubated in 3% O_2_. shRNA-expressing derivative strains were maintained similarly, but in media supplemented with 1 µg/ml or 2 µg/ml puromycin (human and mouse cells, respectively). Serial passaging was performed by resuspending trypsinized cells in fresh DMEM, supplemented as indicated above, and using a dilution factor of 1∶2. For replicative lifespan analyses, we computed the population doubling (PD) level for any given timepoint as [log_2_ (current cell count/(prior cell count/2)) + PD level at prior cell count]. Additionally, we considered a culture to have reached the end of its replicative lifespan (assessed in PDs) when it became incapable of doubling in number (1 PD) within 14 days.

### Mammalian cell stress resistance assays

To assess the relative resistance of mammalian cells to various stresses, we assessed the survival of cells post-stress treatment, as follows. Early passage cells were inoculated into 96 well microtiter plates at a density of 3×10^4^ cells in 100 µL DMEM per well, supplemented and grown as described above. Following a 24 hr incubation period, the media was replaced by DMEM with penicillin/streptomycin and fungizone, but containing 2% bovine serum albumin in place of FBS. Tests for resistance to hydrogen peroxide (500 µM), UV, methyl methanesulphonate (MMS) and heat were performed as previously reported [13], [14], [55]. For assessment of UV resistance, mouse cells were irradiated with 2000 J/m^2^ UVC at 254 nm using a UV Stratalinker 2400 (Stratagene; La Jolla, CA, USA), whereas human cells were irradiated with 500 J/m^2^ at the same wavelength. Mouse and human cells were also subjected to different MMS concentrations (500 µM and 3 mM, respectively). For assessment of heat resistance, human cells were placed on a 42°C heat block for 2 hrs, whereas mouse cells were subjected to 45°C for the same interval. After all treatments, cells were washed with 1× PBS and incubated at 37°C in DMEM supplemented with 2% BSA, antibiotics and fungizone. Survival was measured 18 hr later using the Cell Proliferation Reagent WST-1 kit (Roche; Indianapolis, IN, USA), which gives a quantitative assessment of viable cell number. Four technical replicates were performed for each condition, and all treatments (except for heat shock) were made at 37°C with 3% O_2_ and 6% CO_2_ in air.

### qRT-PCR

For qRT-PCR determination of yeast mRNA and tRNA levels, total RNA was obtained from aged (Day 7) yeast, as described above (*Yeast gene expression profiling*). For determination of *Mtr/MTR*, *RELB* and *NFKBIA* transcript levels in mammalian cells, total RNA was obtained using the RNeasy Mini kit. To assess the abundance of each target RNA, three independent qRT-PCR reactions were performed using the SYBR Green Quantitative RT-PCR kit (Sigma; St. Louis, MO, USA) and a LightCycler 480 (Roche). Relative levels of tRNAs or transcripts of interest were computed and normalized to levels of β-actin (Yeast, *ACT1*; Mouse and human, *Actb*/*ACTB*) using the 2^−ΔΔ*C*^T method [64]. PCR primers used are listed in Table S3.

To assess the significance of differences in tRNA abundance between aged strains, values representing relative tRNA levels were used to perform unpaired two-tailed t-tests. As above, Prism statistical software was used for these analyses.

## Supporting Information

Figure S1 **Restriction of amino acids,** ***per se*** **, does not extend CLS.** (A) Growth in media lacking either valine, isoleucine, threonine, or lysine does not extend CLS, (B) lysine biosynthetic deficiency (genetic lysine restriction) does not extend CLS. Bars denote SEM.
(TIF)

Figure S2 **qRT-PCR determination of relative tRNA levels in aged (Day 7) wild-type and long-lived yeast.** Bars denote SEM.
(TIF)

Table S1 **List of genes differentially-expressed in aged (Day 3) yeast by genetic Meth-R. (See attachment).**
(XLS)

Table S2 **Enrichment of functional annotation categories comprising differentially-expressed genes in aged (Day 3) methionine-restricted yeast cells, dependent on the retrograde response. (See attachment).**
(XLS)

Table S3 **List of primers used for qRT-PCR analyses.**
(XLS)
