# Supplementary figures and images for: Methionine Restriction Activates the Retrograde Response and Confers Both Stress Tolerance and Lifespan Extension to Yeast, Mouse and Human Cells
Source: PLoS One. 2014 May 15;9(5):e97729. doi: 10.1371/journal.pone.0097729 (PMC4022668; doi:10.1371/journal.pone.0097729)

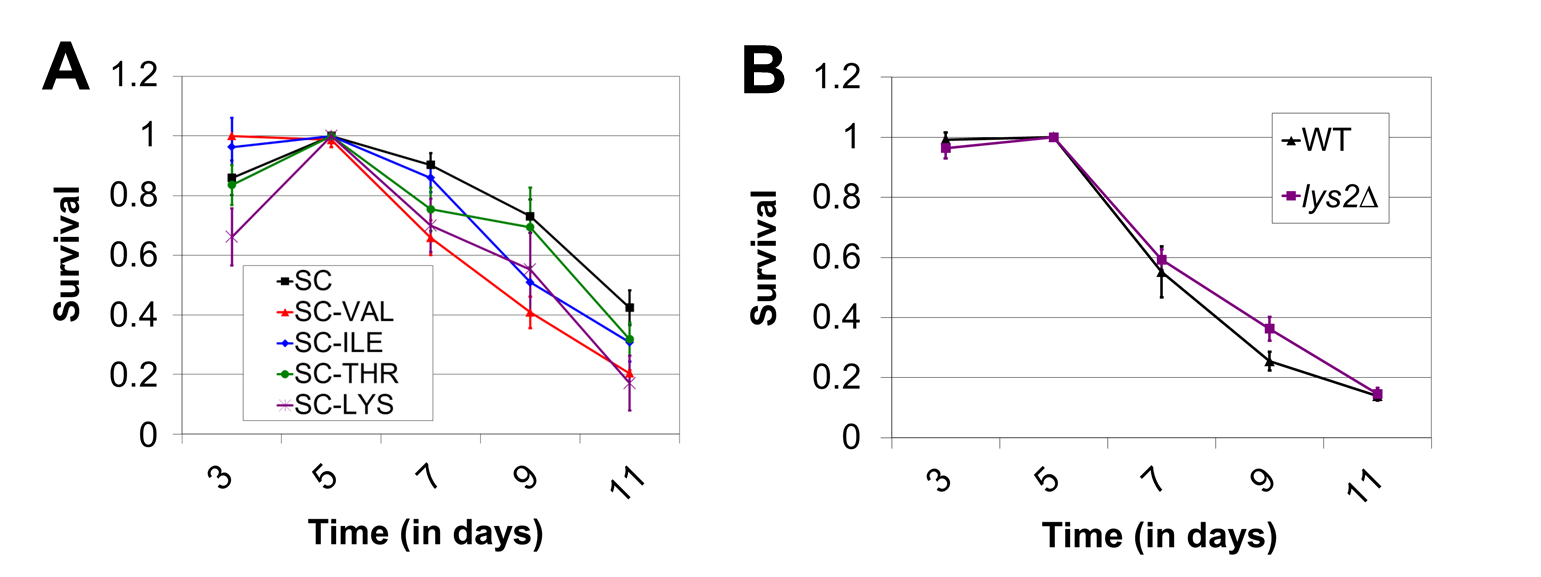

Supplement: Figure S1 — Restriction of amino acids, per se , does not extend CLS. (A) Growth in media lacking either valine, isoleucine, threonine, or lysine does not extend CLS, (B) lysine biosynthetic deficiency (genetic lysine restriction) does not extend CLS. Bars denote SEM. (TIF) [file pone.0097729.s001.tif]

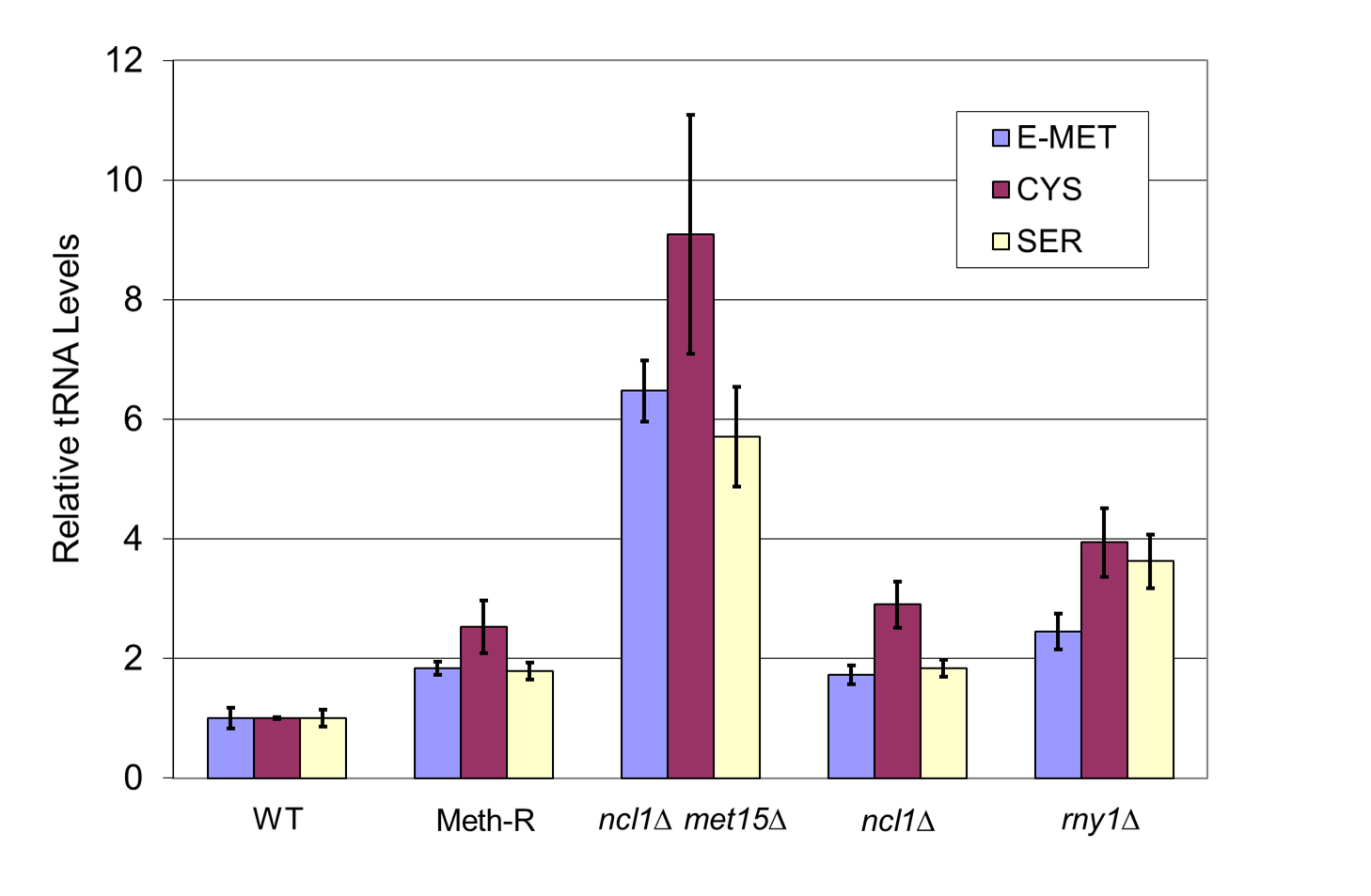

Supplement: Figure S2 — qRT-PCR determination of relative tRNA levels in aged (Day 7) wild-type and long-lived yeast. Bars denote SEM. (TIF) [file pone.0097729.s002.tif]
